# First real-time observation of transverse division in azooxanthellate scleractinian corals

**DOI:** 10.1038/srep41762

**Published:** 2017-02-02

**Authors:** Yuki Tokuda, Hiroko Haraguchi, Yoichi Ezaki

**Affiliations:** 1 Tottori University of Environmental Studies, 1-1-1 Wakabadaikita, Tottori 689-1111, Japan; 2 Tottori Prefectural Museum, 2-124 Higashimachi, Tottori 680-0011, Japan; 3 Research Center for Coastal Lagoon Environments, Shimane University, 1060 Nishikawatsu-cho, Matsue, Shimane 690-8504, Japan; 4 Department of Geosciences, Graduate School of Science, Osaka City University, 3-3-138 Sugimoto, Sumiyoshi-ku, Osaka 558-8585, Japan

## Abstract

Asexual reproduction is one of the most important traits in the evolutionary history of corals. No real-time observations of asexual reproduction in azooxanthellate solitary scleractinian corals have been conducted to date. Here, we describe previously unknown aspects of asexual reproduction by using *Truncatoflabellum spheniscus* (Family Flabellidae) based on observations of transverse division conducted over 1200 days. The findings revealed that (1) transverse division was caused by decalcification; (2) compared to the anthocyathus (upper part of the divided corallum), the soft parts of the anthocaulus (lower part of the divided corallum) were severely damaged and injured during division; (3) these injuries were repaired rapidly; and (4) the anthocaulus regrew and repeatedly produced anthocyathi by means of transverse division. Differences in the patterns of soft-part regeneration and repair, as well as differences in skeletal growth rates between the anthocaulus and the anthocyathus imply that the ecological requirements and reproductive success are different from each other immediately after division. The findings provide important clues for unravelling why asexual reproduction appeared frequently in free-living corals, and the extent to which those modes of reproduction has affected the adaptive and evolutionary success of scleractinian corals throughout the Phanerozoic.

Asexual reproduction plays an important role in corals as it increases the number of individuals and their capacity to adapt to changes in their environment. Modes of asexual reproduction have been examined extensively in extinct and extant colonial corals, especially in terms of the structural and developmental constraints imposed by skeletal development^1^,^2^,^3^,^4^,^5^. Both zooxanthellate and azooxanthellate free-living, whether solitary or colonial, scleractinian corals employ asexual reproduction; occasionally, these modes of asexual reproduction are accompanied by skeletal decalcification (e.g., transverse division, longitudinal division, and anthoblast production)^6^,^7^,^8^,^9^,^10^,^11^,^12^,^13^. Asexual reproduction associated with decalcification in solitary scleractinians has been reported in at least 34 genera in nine families (i.e., Guyniidae, Athemiphylliidae, Dendrophylliidae, Caryophylliidae, Flabellidae, Turbinoliidae, Fungiidae, Poritidae, and Mussidae)^11^,^14^,^15^,^16^. Transverse division in zooxanthellate fungiid corals was initially observed in the 19^th^ century^17^,^18^,^19^. Direct observations in the field and laboratory have revealed that (1) division (disc detachment) is caused by skeletal decalcification, (2) an anthocaulus periodically and repeatedly produces an anthocyathus, and (3) the fungiids show alternations of sexual and asexual generations in their life history^8^,^9^,^20^,^21^,^22^,^23^,^24^. However, no real-time observations of asexual reproduction in azooxanthellate solitary corals have been conducted to date, mainly because of their deep-sea habitats and difficulties associated with sampling and long-term monitoring of living individuals. Consequently, details of the modes of asexual reproduction employed by these corals remain elusive, as studies are typically limited to skeletal morphology (division scars) and structural evidence of rejuvenescence (i.e., regrowth of anthocaulus after transverse division)^6^,^7^,^10^,^25^. In particular, little is known about what happens to the soft parts of corals during division, or the regeneration mechanisms, timing, duration, and cycles of division, and skeletal growth rates before and after division. However, studies on solitary corals of asexual origin have provided invaluable clues for clarifying the various processes associated with asexual reproduction itself and increase in densities (i.e., aggregations in individual corals). Aggregations in reef corals as a result of asexual fragmentation can be found in free-living solitary and/or colonial corals^12^,^16^,^21^,^26^,^27^,^28^.

The genus *Truncatoflabellum* (Family Flabellidae) has a flabellate morphology and several pairs of spines on the corallum edges, although some species do not form spines. *Truncatoflabellum* is exclusively azooxanthellate, solitary, and clonal reproduction occurs by means of transverse division^7^,^29^. The anthocaulus, which is formed by sexual reproduction, gives rise to the anthocyathus (clone individual) by transverse division, which involves decalcification of the skeleton^7^,^29^; the anthocyathus can only reproduce sexually^7^,^25^.

In this long-term study (1200 days), we observed and monitored *Truncatoflabellum spheniscus* in a tank in order to obtain a detailed understanding of the structural processes associated with transverse division, especially aspects related to the division and repair of soft and skeletal parts of the anthocaulus and anthocyathus, respectively. In addition, skeletal growth rates were estimated in each anthocaulus and anthocyathus and the mode of transverse division in *Truncatoflabellum* was interpreted within the context of the adaption and evolution of free-living scleractinian corals.

## Results

### Transverse division

**Figure 1:**
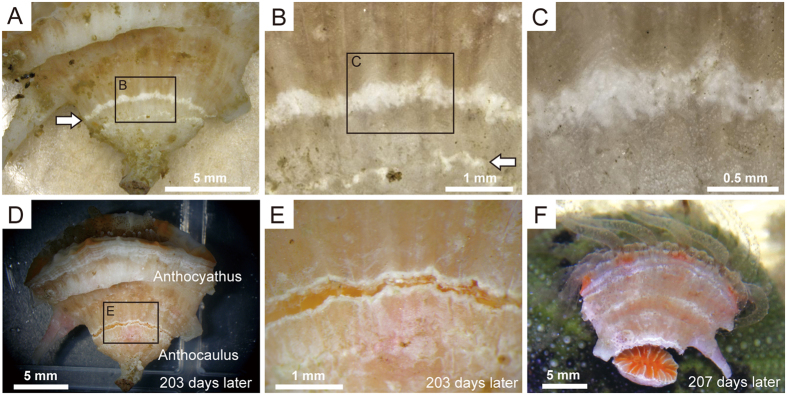
Decalcification features of the anthocaulus stage of *Truncatoflabellum spheniscus*. (**A–C**) Decalcification (white band) on the wall surface just below the level of lateral spines. (**A**) Lateral view of a corallum and decalcification indicated by a white arrow. (**B**) Enlargement of the black rectangle in (**A**) to show the decalcification band along growth lines of the wall. Decalcification and rejuvenescence associated with a previous transverse division (white arrow). (**C**) Enlargement of the black rectangle in (**B**) to show area of decalcification consists of circular to irregularly shaped, beaded pores. (**D,E**) Decalcification extended all the way around the skeleton of the anthocaulus stage, 203 days after (**A–C**). (**D**) Gap separating the upper and lower parts of the corallite. (**E**) Enlargement of the black rectangle in (**D**). (**F**) Part of the anthocyathus stage that has fallen over under its own weight, 207 days after (**A–C**).

**Figure 2:**
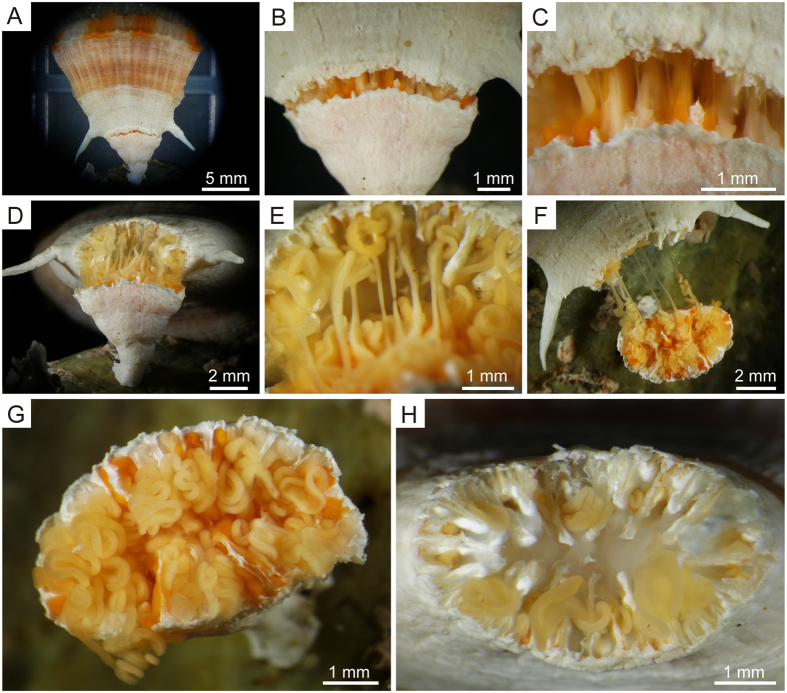
Processes associated with division of the soft parts of *Truncatoflabellum spheniscus* after skeletal decalcification. (**A**) Lateral view of entire corallum. (**B**) Enlargement of the divided stages shown in (**A**). (**C**) Enlargement of the divided stages shown in (**B**), thin membranes and mesenterial filaments were stretched due as the anthocyathus stage separated from anthocaulus stage under its own weight. (**D**) Stretched and torn mesenterial filaments. (**E**) Enlargement of the divided area shown in (**D**). (**F**) Calical view of the anthocaulus stage. Severely torn mesenterial filaments. (**G**) Calical part of anthocaulus stage immediately after the division, with extensive damage to the soft tissues and numerous mesenterial filaments can be seen. (**H**) Basal part of the anthocyathus stage immediately after the division, showing mesenterial filaments extending from pits.

The process of transverse division in mature anthocauli was initiated by decalcification of the calical (inner) parts of walls, immediately below the level of the lateral spines and at neighbouring septa. As decalcification progressed, the parts of the wall that had lost its luster and appeared as a white band measuring 0.1–0.2 mm on the corallum surface (Fig. 1A–C). Circular and irregularly shaped beaded pores subsequently appeared between the septa on the decalcified areas of corallum walls. Once decalcification had proceeded around the full circumference of the anthocaulus, a gap developed, separating the upper (anthocyathus) and lower (anthocaulus) parts of the corallites, which remained connected to each other by their soft parts alone (Figs 1D,E and 2A–C). Unable to support the weight of the upper part of the corallum (anthocyathus), the thin membranes and mesenterial filaments constituting the soft parts stretched and tore free from the lower part of the corallum (anthocaulus) (Figs 1F and 2D–F).

**Figure 3:**
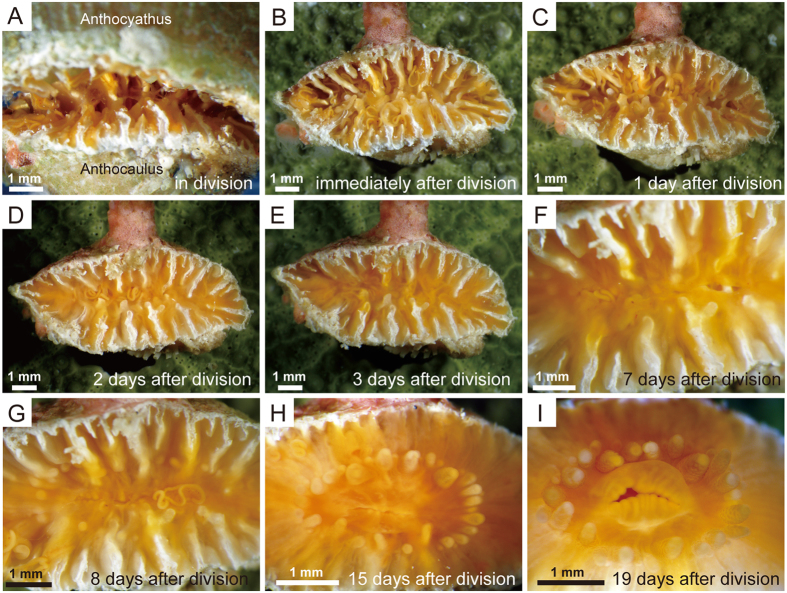
Polyp regeneration of the anthocaulus stage after transverse division in *Truncatoflabellum spheniscus*. (**A**) Separation of the anthocyathus stage from the anthocaulus stage. (**B**) Calical part of anthocaulus stage immediately after division, showing extensive damage to the soft tissues. (**C**) 1 day after division; sheet-like tissues formed along the periphery of the calice. (**D**) 2 days after division; sheet-like tissues spread near the centre of the calice, with the uppermost parts of septa partly covered by soft tissues. (**E**) 3 days after division; sheet-like tissues extended further, completely covering the polyp in the calice. (**F**) 7 days after division; two slits formed independently at the centre of oral disc parallel to the two directive septa. Tentacle buds formed around slits. (**G**) 8 days after division; a mouth was regenerated from an opening within the oral disc between the two slits. (**H**) 15 days after division, the mouth and its surrounding muscle system were regenerated. (**I**) 19 days after division, tentacles with acrospheres at their tips were regenerated.

Immediately after dividing, the uppermost peripheries of the septa and the wall of the anthocaulus, which were not yet covered by soft tissues, turned white due to decalcification (Figs 2G,H and 3A,B). Deeply incised hollow structures attributable to decalcification were observed at the central parts of septa (i.e., rapid accretion deposits^30^) (Figs 2G and 3A,B). Columellae did not form in the centre of the calice, where soft tissues remained (Figs 2G and 3B). The soft tissues were severely damaged and there were no mouths and tentacles in the anthocaulus (Figs 2G and 3B). However, mesenterial filaments were recognized in the calice (Figs 2G and 3B). Immediately after division, the height, greater calicular diameter, and lesser calicular diameter of the anthocauli were 6.22 ± 0.5 mm, 7.89 ± 0.5 mm, and 4.21 ± 0.1 mm, respectively (mean ± SE; N = 8).

**Figure 4:**
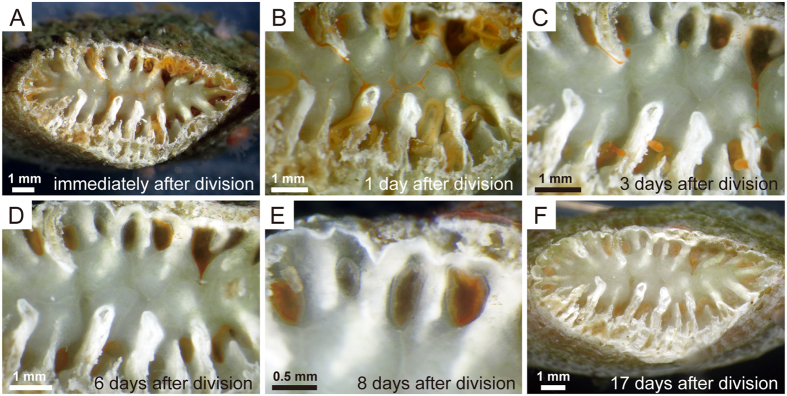
Repairs of divided part of the anthocyathus stage of *Truncatoflabellum spheniscus*. (**A**) Basal part of the anthocyathus stage immediately after division, showing mesenterial filaments extruding from several pits. (**B**) 1 day after division; several pits were covered with thin soft tissue. (**C**) 3 days after division; all pits were covered with soft tissues. (**D**) 6 days after division; thin horizontal skeletal elements developed around the rims of the pits. (**E**) 8 days after division; pit-infilling proceeded gradually toward the centre. (**F**) 17 days after division; all pits were completely occluded by skeletal partitions.

In the basal part of the anthocyathus, the peripheries of septa and inner sides of walls were covered with thick stereome (i.e., thickening deposits^30^) (Figs 2H, 4A,B). A complete set of first (S1) and second cycles of septa (S2), along with eight third-cycle septa (S3) were fused axially to form a columella (Figs 2H, 4A). In some cases, S4 septa could also be recognized at the calicular base of the corallum. Decalcification was restricted to parts that were originally septa (Fig. 4), and no traces of decalcification were observed in the columella or on the thick stereomes (i.e., thickened deposits) on septa and walls (Fig. 4). Stereomes were observed on parts of septa, sometimes as early as immediately after division (Fig. 4A,B). At the basal region of the anthocyathus, 20 to 24 pits encircled by septa, walls, and columella were infilled with soft tissues (Figs 2H and 4A); at the upper part of anthocyathus, relatively little damage occurred and the polyp was still capable of extending its tentacles, even immediately after division (Fig. 1F).

### Regeneration of the anthocaulus polyp

The extensive tissue damage associated with transverse division in the anthocaulus meant that polyps had no mouth, tentacles or oral disc (Figs 2G and 3B). One day after division, sheet-like tissues formed along the periphery of the calice; no oral disc was present in the centre of the calice but numerous mesenterial filaments were present (Fig. 3C). Two days after division, these sheet-like tissues extended to near the centre of the calice, and the uppermost part (hereafter referred to as divided part) of the septa was partly covered with soft tissue (Fig. 3D). Three days after division, the sheet-like tissues had extended further and completely covered the part of the calice where the polyp resides, preventing mesenterial filaments from protruding beyond the sheet-like tissues (Fig. 3E). Seven days after division, two slits formed independently in the centre of oral disc parallel to two directive septa (Fig. 3F). Eight days after division, a mouth formed from an opening in the oral disc between the two slits (Fig. 3G). Small knob-like protrusions (hereafter referred to as tentacle buds) were scattered around a mouth. The anthocaulus polyp was able to open and close the mouth to capture food by moving the mesenterial filaments instead of tentacles. In a marked contrast to the marginal parts of septa, the axial parts of septa were mostly enveloped by soft tissues (Fig. 3G). By 15 days after the transverse division, the mouth and its surrounding musculature had regenerated completely, and more than 30 tentacles surrounded the oral disc (Fig. 3H). The tentacle tips became knobbed and were shaped like acrospheres (i.e., thickened tentacle tips containing batteries of nematocysts, which may show an aberrant pigmentation). Nineteen days after the division, acrospheres had regenerated at the tentacle tips and tentacles were able to capture food without extending laterally (Fig. 3I). Septa extended upward along the same axis as preexisting septa, and rejuvenescence was observed in the walls, which started to form upon inner portions of divided ones.

### Repairs of divided part of anthocyathus

Immediately after the transverse division, 20 pits bordered by septa, walls, and columella penetrated into the interior of the anthocyathus resulting in extensive soft tissue damage at the basal part of the anthocyathus (Figs 2H and 4A). Mesenterial filaments extruded from several pits and columella and stereome surfaces were covered with films of soft tissue (Fig. 4A). One day after the transverse division, several pits were covered by a thin layer of soft tissues, while the remaining pits had mesenterial filaments extruding from them. Thin, thread-like soft tissues, which formed from the thin soft tissues covering the columella and stereome, formed a network of threads and were connected to tissues in the pits (Fig. 4B). Three days after division, all of the pits were covered with soft tissues, some of which had the appearance of tangled clumps and were derived from the thread-like soft tissues (Fig. 4C). Six days after division, thin horizontal skeletons were observed along the outer rims of the pits (Fig. 4D). Eight days after division, the pit-infilling process progressed gradually along the plane of the thin soft tissues toward the pit centre (Fig. 4E), and by 17 days after the transverse division, all of the pits were completely occluded by skeletal partitions (Fig. 4F).

### Regrowth of anthocaulus skeletons

**Figure 5:**
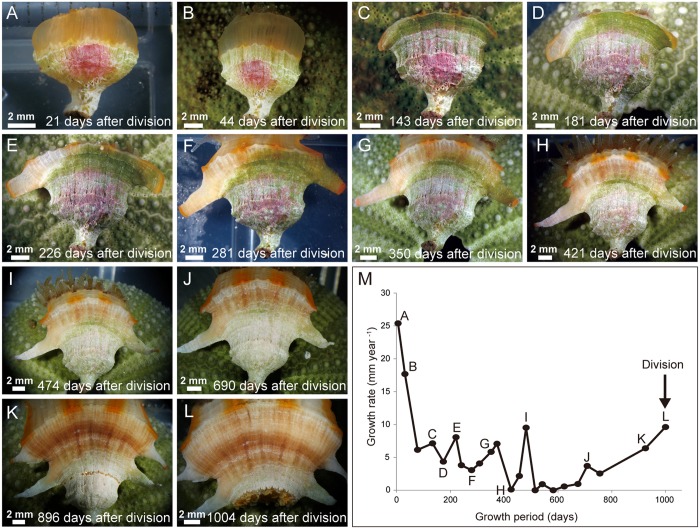
Skeletal regrowth and linear growth rates of the anthocaulus stage of *Truncatoflabellum spheniscus* after transverse division. (**A**) Lateral view of an anthocaulus stage 21 days after division. The regenerated wall grew upward with its maximal calicular diameter almost unchanged. (**B**) 44 days after division. (**C**) 143 days after division, directions of the wall are drastically changed at both edges of a corallum. (**D**) 181 days after division. (**E**) 226 days after division, hollow tube-like structures formed from the spines. (**F–H**) both wall and spines grew independently. (**I**) 474 days after division, a white decalcification band appeared on the outer surface of the anthocaulus wall, just below the level of lateral spines. (**J**) 690 days after division. (**K**) 896 days after division, the decalcified part on the wall surface almost disappeared. (**L**) 1004 days after division, next transverse division occurred. (**M**) Variation in linear growth rate of the anthocaulus after transverse division.

Six instances of transverse division in *Truncatoflabellum* were monitored meticulously. Approximately 15 days after division, the walls extended upward from the divided parts in a process referred to as rejuvenescence. The regenerated walls grew upward, with the greater calicular diameter remaining almost unchanged in the early stages of regrowth (Fig. 5A,B). About 150 days after division, the direction of wall growth changed markedly, especially the edges of the corallum, leading to the formation of a pair of spines (Fig. 5C,D). Approximately 200 days after division, the spines developed into hollow tube-like structures, which subsequently developed separately from the wall (Fig. 5E–G). The spines, which had very acute tips, ceased growing approximately 400 days after division (Fig. 5H). At approximately 500 days after division, a white band characteristic of decalcification appeared along the outer surface of anthocaulus walls just below the level of lateral spines (Fig. 5I). Approximately 700 days after division, decalcification progressed and the direction of wall growth changed drastically to form new pair of lateral spines (Fig. 5J). Approximately 900 days after division, the decalcified part of the wall surface almost disappeared and decalcification was completed (Fig. 5K). The next transverse division occurred at approximately 1000 days after division (Fig. 5L). The mean time interval between the development of transverse divisions was 982 ± 134 days (N = 3).

### Skeletal growth rates

**Figure 6:**
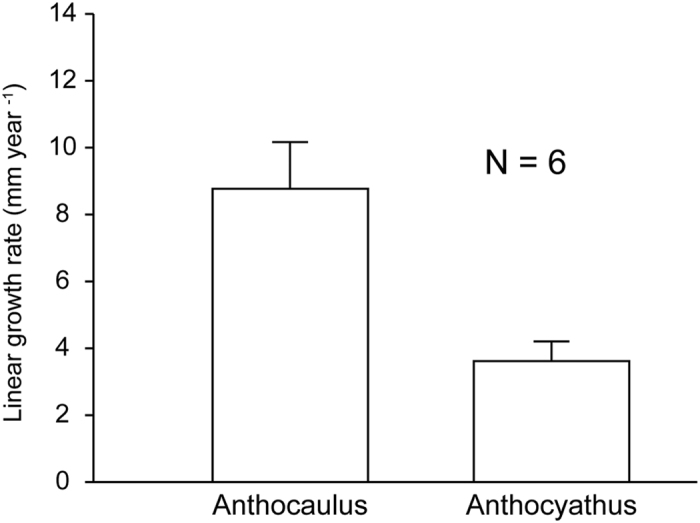
Variations in linear growth rate between the anthocaulus and anthocyathus stages after transverse division.

The linear growth rates for anthocauli and anthocyathi were 8.8 ± 1.4 and 3.6 ± 0.6 mm yr^−1^, respectively (N = 6; Fig. 6). Analysis by paired *t*-tests revealed significant differences (P < 0.01) between the linear growth rates for anthocauli and anthocyathi. The growth rate for the repaired parts at the basal scar of anthocyathi was 8.9 ± 1.0 mm yr^−1^ (mean ± SE; N = 4).

**Figure 7:**
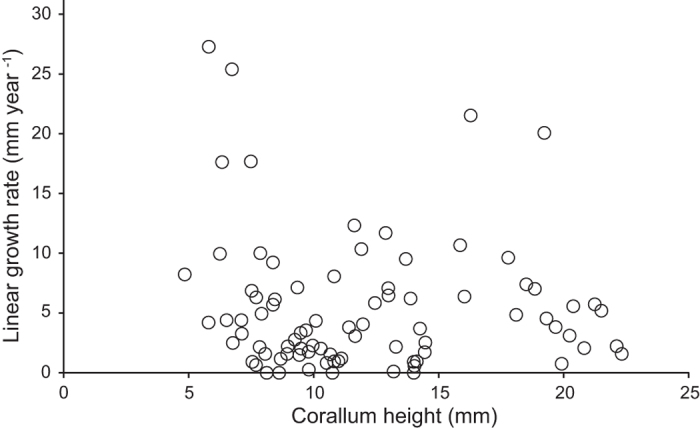
Variations in linear growth rate of the anthocauli of *Truncatoflabellum spheniscus*. This plot corresponds to a Gulland and Holt plot.

Growth rates were obtained for the full size range of *Truncatoflabellum spheniscus* anthocauli (Fig. 7). The growth rates for the anthocauli varied according to coral height. Thus, individuals measuring 4–8 mm in height grew 8.7 ± 1.9 mm yr^−1^ (N = 18), individuals measuring 8–12 mm in height grew 3.3 ± 0.6 mm yr^−1^ (N = 32), individuals measuring 12–16 mm in height grew 3.7 ± 0.9 mm yr^−1^ (N = 16), and individuals measuring 16–20 mm in height grew 13.7 ± 3.0 mm yr^−1^ (N = 5).

## Discussion

Real-time observations of the transverse divisions in the azooxanthellate scleractinian *Truncatoflabellum spheniscus* revealed the following: (1) transverse division was caused by decalcification; (2) compared to the anthocyathus (upper part of divided corallum), the soft parts of the anthocaulus (lower part of divided corallum) were severely damaged and injured at the time of the division; (3) these injuries were repaired rapidly; and (4) the anthocaulus quickly regrew and repeatedly produced anthocyathi by transverse division.

Decalcification of *Truncatoflabellum* was initiated on the septa and the calical sides of walls (Fig. 1A–C). The observed decalcification processes were very similar to those reported in fungiid corals^8^,^11^,^13^,^20^,^22^,^23^,^24^. The anthocyathus of *Truncatoflabellum* has a thick stereome and columella at the aboral base immediately above the site where decalcification occurs (Figs 2H and 4A). Differences in skeletal characteristics (skeletal thickening patterns) were observed between the anthocaulus and anthocyathus stages immediately after division (Fig. 2G,H), implying that skeletal formation had already been initiated before division. The extent of the soft parts connecting the anthocyathus to the anthocaulus was also limited, which meant that the damaged parts on the aboral bases of the anthocyathus stage could be repaired quickly after division (Fig. 4). In addition, the mesenteries, coelenteron, and other essential organs were elevated by the development of thickened deposits, which enable the anthocyathus to continue feeding, protect itself and produce gametes after division. The anthocyathus is considerably larger than the anthocaulus at the time of division. The flabellate morphology and large surface area of *Truncatoflabellum* prevents the coral from sinking into soft substrates^25^. Similarly, the increased thickening deposits in the lower parts of anthocyathus might contribute towards an increase in stability in substrates, as well as keeping the calice oriented towards the sea surface which would be advantageous for its food acquisition, as seen in other free-living corals^31^,^32^,^33^. The skeletal structure of the anthocyathus therefore appears to have a reproductive as well as an ecological function that facilitates free-living on soft substrates.

In contrast, thickening deposits on the septa and calicular surfaces of the walls of the anthocaulus stage is very thin (Figs 2G and 3B), implying that the anthocaulus has sufficient space for soft tissues in the calice. Unlike the severely injured polypal parts of the anthocaulus (Figs 2G and 3A,B), the presence of calical tissues and spaces in the anthocaulus calice would facilitate the efficient regeneration of the polyp and accommodate the coelenteron after division^34^,^35^. By 3 days after division, sheet-like tissues completely covered the damaged parts of the polyp in the calice (Fig. 3E). Furthermore, a mouth was regenerated after only 8 days to capture food by moving the mesenterial filaments (Fig. 3G), suggesting the presence of a digestive coelenteron before the appearance of tentacles. In polyps of the starlet sea anemone, *Nematostella vectensis*, wound closure occurs very soon after budding^36^. However, in *Truncatoflabellum*, rapid wound closure is difficult because the soft parts are attached to the skeletal surface by desmocytes^37^. It is possible that the anthocaulus of *Truncatoflabellum* uses the sheet-like tissues for wound closure and the mesenterial filaments for protection, albeit temporarily. The tentacles formed from knob-like protrusions 7 days after division (Fig. 3G–I). This was similar to tentacle regeneration in *N. vectensis*, in which budding occurs perpendicular to the primary body axis^36^. Regeneration in *Fungia fungites* occurs as follows: a detachment scar of stalk was covered rapidly by regenerating soft tissues almost within one day; one week later, skeletons were secreted with no complete mouth and tentacles^8^. Although *Fungia* and *Truncatoflabellum* belong to different families^38^, the regeneration processes are similar.

Compared to the polyp of the anthocyathus, which was not severely damaged during and after division and continued to expand, the polypal tissues of the anthocaulus stage were severely damaged (Fig. 1F). These observations suggest that damage allocation associated with transverse division in *Truncatoflabellum* differs between the anthocyathus and anthocaulus, with the anthocaulus (asexual phase) taking precedence over the anthocyathus (sexual phase). These modes of transverse division are totally different from those of longitudinal division, in which the offsets inherit the tentacles and a mouth from their parents. A trade-off relationship between regeneration and reproduction has been suggested for many colonial corals^39^,^40^. In the anthocyathus of the solitary scleractinian, *Fungia granulosa*, sexual reproduction is favoured if energetic and cellular reserves are insufficient for both tissue repair and reproduction^34^. However, the life cycle of *Truncatoflabellum* is characterized by the distinct alternation of generations (asexual phase in anthocauli vs. sexual in anthocyathi)^7^,^25^. It is therefore considered that an anthocaulus of *Truncatoflabellum* will use all of its energetic and cellular reserves to regeneration during and after transverse division without attempting sexual reproduction. This method of energy allocation in the anthocaulus stage is considered to result in efficient polypal repair and growth following transverse division, even if the polypal tissues are extensively damaged for a time. In contrast, the anthocyathus experiences considerably less tissue damage during the transition from the previous anthocaulus. The anthocyathus can therefore expend considerably more energy on sexual reproduction as it minimizes the extent of regeneration. The alternation of generations is considered to be one of the best ways to solve reproduction-regeneration trade-off problems in damaged corals that employ transverse division as part of reproduction.

The skeletal growth rate for the anthocyathus of *Truncatoflabellum spheniscus* was similar to that for other azooxanthellate scleractinians and medium- to larger-sized individuals of *Flabellum alabastrum* (Family Flabellidae)^41^,^42^,^43^,^44^,^45^. Conversely, the growth rate for the anthocaulus was similar to those for the smallest individuals of *F. alabastrum*, and higher than that for other azooxanthellate scleractinians^45^. In scleractinian corals, growth rates decrease with age^28^,^44^,^45^,^46^,^47^. However, after transverse division, the rate of wall growth in anthocauli is higher than it is in anthocyathi over the same period of time (Fig. 6). To the best of our knowledge, this is the first study to provide evidence of restoration of skeletal growth rates in solitary corals. The accelerated growth rates for anthocauli after the division lead to a shortening of the time intervals to the next division. In addition, higher rates of pit-infilling in the basal part of anthocyathi skeletons facilitate rapid tissue repair after division. The rates of wall growth in the anthocauli and pit-infilling in the skeletons of anthocyathi both have a marked influence on the efficiency of tissue repair following transverse division and the survival rates of divided individuals.

The patterns of both upper skeletal formation (i.e., the future anthocyathus after transverse division) and lower skeletal formation (i.e., the future anthocaulus after division) in the anthocaulus of *Truncatoflabellum*, as well as the skeletal growth rates after transverse division for the anthocaulus and anthocyathus are different. The differences in the modes of skeletal development before and after transverse division play an important part in subsequent regeneration by decreasing the time taken for asexual reproduction and facilitating adaptation to a free-living mode of life. Strict control of the sequence of calcification, decalcification, polyp regeneration during and after transverse division in *Truncatoflabellum* is therefore necessary to increase the number of clonal individuals efficiently. Therefore, the system of asexual reproduction in solitary corals is considered to have been closely linked to the evolution of modes of skeletal growth.

It is noteworthy that the essential process of the transverse division (i.e., decalcification, polypal damage and rapid repair after division, and skeletal regrowth) of azooxanthellate *Truncatoflabellum* is almost the same with the transverse division of zooxanthellate fungiid corals, although both groups require different habitat conditions (e.g., depth, light, temperature, and others). *Truncatoflabellum* has evoloved from an ancestor without transverse division^48^,^49^. Moreover, attached coral *Rhizotrochus*, which is a sister group of *Truncatoflabellum*, has lost both the free-living mode of life and the ability of transverse division in flabellid evolutionary pathway^48^,^49^. Similarly, some fungiid corals independently lost the ability of transverse division associated with decalcification and free-living mode of life (e.g., *Cycloseris mokai, C. explanulata, C. explanulata, C. wellsi, Lithophyllon undulatum*)^15^,^50^. An ability of decalcification are thus independently appeared and lost in each scleractinian clade. Moreover, *Fungia fungites* does not have specialized cells for skeletal dissolution in the vicinity of dissolving skeletons at the time of transverse division^20^. In contrast, calicoblastic cells in the epithelium above dissolving skeletons are still metabolically active, and are therefore closely involved in the resorption of skeletons^20^. This suggests that the decalcification process might result from subtle modifications of calcification process, which is commonly provided with scleractinian corals (e.g., restriction of Ca^2+^-ATPase, which actively remove protons from the calcifying fluid, in calicoblastic epithelium cells^51^). *Truncatoflabellum* evolved from a solitary coral having a free-living mode of life without transverse division^48^,^49^. Its anthocyathus is thus adaptive in advance to soft substrates. 330 species of azooxanthellate scleractinian corals, or 22% of all scleractinians, live on deep-water sandy and/or muddy substrates^52^. Moreover, 65 species of the 330 free-living species experience transverse division^52^. The free-living mode of life consequently occurs by the division. Therefore, adaptation to the soft substrates is essential to the evolution of asexual reproduction with skeletal decalcification. This might be one of the reasons why asexual reproduction (transverse division in this case) associated with skeletal decalcification evolved frequently in several free-living coral clades.

This study clarified that transverse division occurs in several scleractinian corals through a series of highly efficient procedures. Those findings provide important clues for unravelling why asexual reproduction appeared frequently in azooxanthellate solitary corals, and the extent to which those modes of reproduction has affected the adaptive and evolutionary success of scleractinian corals throughout the Phanerozoic.

## Materials and Methods

Six anthocaulus specimens of *Truncatoflabellum* were collected off Koshiki-jima islands at depths of 50 to 100 m in July 2011. The corals were observed for more than 1200 days in a 1000 L tank containing filtered seawater at a temperature of 17 to 19 °C. The corals were maintained under dark conditions and fed twice a week with frozen copepods. Anthocaulus-stage specimens, which were attached to pebbles and set upon a spherical stage (echinoid test) in order to reconstruct natural conditions as far as possible. Anthocyathus specimens are placed on plastic mesh plates, such that they lay on their flattened sides. The specimens were placed sufficiently apart to prevent their tentacles contacting each other. Details of macro-skeletal features were observed using an optical microscope (SMZ-1500; Nikon, Japan) and a digital microscope (VHX-1000; Keyence, Japan).

Skeletal growth was measured as vertical rate of extension over 1200 days and photographs were taken at monthly or bimonthly intervals under a stereomicroscope (Olympus SZX7) equipped with a digital camera. Photographs were always taken at the same distance and at the same angle. Linear growth were quantified using ImageJ software (National Institutes of Health, http://imagej.nih.gov/ij/)^53^. Images were calibrated using a distinct natural marking. Linear growth rates were normalized to 1 yr. The linear growth rates of six anthocaulus specimens were estimated by the Gulland and Holt method^54^, and were divided 4 size classes in 4–8 mm in height (N = 18), 8–12 mm (N = 32), 12–16 mm (N = 16), 16–20 mm (N = 5).

## Additional Information

**How to cite this article:** Tokuda, Y. *et al*. First real-time observation of transverse division in azooxanthellate scleractinian corals. *Sci. Rep.* **7**, 41762; doi: 10.1038/srep41762 (2017).

**Publisher's note:** Springer Nature remains neutral with regard to jurisdictional claims in published maps and institutional affiliations.
